# Fine−Needle Aspiration Biopsy in the Diagnosis and Follow−Up of Thyroid Nodules in Childhood

**DOI:** 10.4274/jcrpe.v2i2.78

**Published:** 2010-05-05

**Authors:** Ayça Altıncık, Korcan Demir, Ayhan Abacı, Ece Böber, Atilla Büyükgebiz

**Affiliations:** 1 Dokuz Eylül University Faculty of Medicine, Pediatric Endocrinology, İzmir, Turkey; 2 Keçiören Training and Research Hospital, Pediatric Endocrinology, Ankara, Turkey; 3 Bilim University Faculty of Medicine, Pediatric Endocrinology, İstanbul, Turkey; +90 232 243 63 99ayca.altincik@deu.edu.trDokuz Eylül University Faculty of Medicine, Pediatric Endocrinology, İzmir, Turkey

**Keywords:** Thyroid nodules, fine−needle aspiration biopsy, FNAB, children, thyroid carcinoma

## Abstract

**Objective**: To assess the role of fine−needle aspiration biopsy (FNAB) in the management of pediatric thyroid nodules.

**Methods**: Results of 30 FNABs performed in our clinic were retrospectively reviewed. Clinical and surgical follow−up data were obtained from the patient files, and clinical correlation and accuracy of FNAB were evaluated.

**Results**: The results of 30 FNABs were reported as benign in 24 (80%), insufficient in 4 (13.3%) patients, malignant in 1 (3.3%), and suspicious in 1 (3.3%) patient. One patient with a FNAB result of malignancy underwent surgery and the histological diagnosis was papillary carcinoma. FNAB was repeated in two of the insufficient biopsies, and reported as benign; in one of these patients, the thyroid nodule disappeared and in one, remained stable at clinical follow−up. Four of the patients with benign FNAB results underwent surgery at clinical follow−up because of an increase in the size of the nodules and one patient was found to have papillary carcinoma. The remaining patients were clinically followed. In this study, the malignancy prevalence was 6.6% in patients with thyroid nodules. There was only one falsenegative

case.

**Conclusion**: FNAB is a reliable diagnostic tool in the management of pediatric thyroid nodules.

**Conflict of interest:**None declared.

## INTRODUCTION

Thyroid nodules are rare in children when compared to adults and the prevalence increases with age, ranging between 0.05 and 1.8% (1, 2, 3). Risk factors for development of thyroid nodules in children include head and neck irradiation, female sex, iodine deficiency, family history of thyroid disease, previous or coexisting thyroid disease (4).

Thyroid nodules commonly present as an asymptomatic thyroid mass. The differential diagnosis of a thyroid nodule includes adenoma, colloid nodule, thyroglossal duct cyst, lymphocytic thyroiditis and malignancies. Although the majority of thyroid nodules are benign, the risk of malignancy is higher in children than in adults and is reported in a wide range as 5−50% (5, 6, 7). Thus, differentiating malignancy from benign lesions is the most challenging dilemma for physicians.

Physical examination findings, family history, laboratory parameters such as thyroid function tests and thyroid antibodies, thyroglobulin, ultrasonography (US) and scintigraphy are methods used in the diagnosis. However, the accuracy of these clinical and imaging procedures is less than that of the cytological examinations (8, 9). Fine−needle aspiration biopsy (FNAB) has been established as a useful test in the differentiation of a nodule and is the most accurate method suggested by the American Thyroid Association (10). There is a lack of consensus regarding the use of FNAB in pediatric patients and the literature is controversial. Many studies have found that FNAB is a highly sensitive and accurate test in differentiating benign lesions from malignant nodules and that it decreases the need for surgery (8, 9, 11, 12). On the other hand, some studies report that FNAB is not an accurate tool because of its poor sensitivity and underlined the need for more radical procedures for definitive diagnosis (13, 14).

## METHODS

The records of 30 children, who attended the Pediatric Endocrinology division at Dokuz Eylül University Faculty of Medicine and underwent FNAB for thyroid nodules in the past 13 years, were retrospectively studied.

FNAB was performed by a pediatric endocrinologist in cases of palpable, single nodules larger than 1.0 cm in diameter. FNABs of non−palpable, multiple nodules and of those with a diameter less than 1.0 cm were performed by a radiologist under ultrasound guidance. In the presence of multiple nodules, evaluation for the selection of the nodule requiring cytological examination was performed by the radiologist.

Following administration of a local anaesthetic, 18−22 gauge needles attached to 10−20 mL syringes were used to obtain the samples. One to five separate aspirates were collected in each case. The cytological material was smeared onto slides. Approximately half of the slides were immediately fixed with 95% ethyl−alcohol and stained with Papanicolaou stain. Other slides were air−dried to be stained with Wright−Giemsa. Aspirates were considered benign when the specimens contained colloid material, macrophages and aggregates of normal−appearing uniform thyroid cells. Aspirates were considered insufficient when only cyst fluid or few cells were identified, suspicious when a cellular follicular lesion or a follicular neoplasm was encountered, and were diagnosed as malignant when papillary carcinoma was identified.

Clinical follow−up data of the patients were obtained from the patient files. The thyroid FNAB results were classified as malignant, benign, suspicious or insufficient.

## RESULTS

The study group consisted of 4 (13.3%) male and 26 (86.7%) female patients with a mean age of 14.6 (range, 8.5−19.5) years. Seven of the FNABs were performed by a pediatric endocrinologist, and the remaining 23 were done by a radiologist.

Seventeen patients had a single nodule, while 13 had multiple nodules. The single nodules were all hypoechoic, except one with heterogeneous content. Four of the 13 patients with multiple nodules had hypoechoic−cystic nodules, two had anechoic nodules and the remaining patients had mixed multiple nodules (some of the nodules were hypoechoic and some isoechoic).

The cytological classification of the FNAB results showed that 24 (80%) of the aspirates were diagnosed as benign, 4 (13.3%) were considered as insufficient, 1 (3.3%) as malignant, and 1 (3.3%) as suspicious. Re−aspiration under US guidance was done in two patients evaluated as having insufficient cytology and were reported as benign. The other two patients with insufficient cytology were followed clinically; the nodule disappeared in one patient and remained stable in the other one. The patient showing suspicious cytology was a 16−year−old girl and had multiple hypoechogenic nodules in the left lobe. Cytological examination of the reaspirated material in this patient revealed distinct hypercellularity and left hemithyroidectomy was performed. The histological diagnosis was benign. After three−year follow−up period, she was transferred to adult endocrinology.

In the two patients with FNAB results reported as papillary carcinoma and adenoma, and who underwent surgery, the histological diagnoses were compatible with the FNAB results. Benign nodules were clinically followed for a mean period of 21.7 (range, 3−72) months. At the end of the clinical follow−up, a 50% decrease in nodule dimensions was observed with thyroid hormone suppression in 10 of the 24 benign nodules and these patients were followed clinically. Five of the remaining 14 benign nodules underwent surgery when the number and/or the dimensions of the nodule increased. One of these patients was diagnosed as having papillary carcinoma, while the lesions were identified as benign in 4. All remaining patients were followed clinically. FNAB and the clinical results at follow−up are shown in Table 1.

**Table 1:**
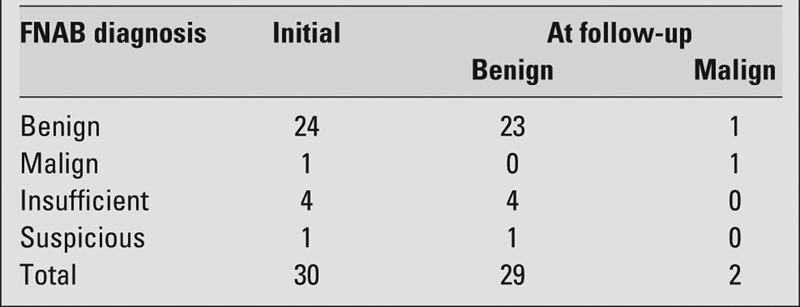
Histological or clinical diagnosis of nodules at presentation and at follow−up

## DISCUSSION

Thyroid nodules are rare in the pediatric population (2, 3). Although the majority of the nodules are benign, risk of malignancy is higher in childhood than in adults (2, 7). In the differential diagnosis of a thyroid nodule, follicular adenoma and colloid goiter are the most frequently reported benign etiologies (8, 9, 13, 15). In various studies, papillary carcinoma was found to be the most frequent (5−47%) malignancy in pediatric cases with thyroid nodules (6, 8, 9, 11, 16, 17). The reasons for this large discrepancy in the rate are attributed to factors such as differences in diagnostic procedures, ethnic origin of the study groups and reluctance to use unnecessary neck and head radiation. In our study, similar to the results reported by others (9), the majority of FNAB findings revealed benign lesions.
